# Supplementary material for: Decoding of the ubiquitin code for clearance of colliding ribosomes by the RQT complex
Source: Nat Commun. 2023 Jan 10;14:79. doi: 10.1038/s41467-022-35608-4 (PMC9831982; doi:10.1038/s41467-022-35608-4)
Supplement: Supplementary file 1 — Supplementary Information [file 41467_2022_35608_MOESM1_ESM.pdf]

Supplementary information

Decoding of the ubiquitin code for clearance of colliding ribosomes by the RQT complex

Authors: Yoshitaka Matsuo[1,*] Takayuki Uchihashi[2-4], and Toshifumi Inada[1,*]

Affiliations:

[1]Division of RNA and Gene regulation, Institute of Medical Science, The University of Tokyo, Tokyo 108-8639, Japan

[2]Department of Physics, Nagoya University, Nagoya 464-8602, Japan

[3]Institute for Glyco-core Research (iGCORE), Nagoya University, Nagoya, Japan

[4]Department of Creative Research, Exploratory Research Center on Life and Living Systems, National Institutes of Natural Sciences, Okazaki, Japan

*Correspondence: yoshitaka-matsuo@g.ecc.u-tokyo.ac.jp (Y.M.)
toshiinada@ims.u-tokyo.ac.jp (T. I.)

This PDF file includes:

Supplementary Figs 1 to 7

Supplementary Table 1 to 4

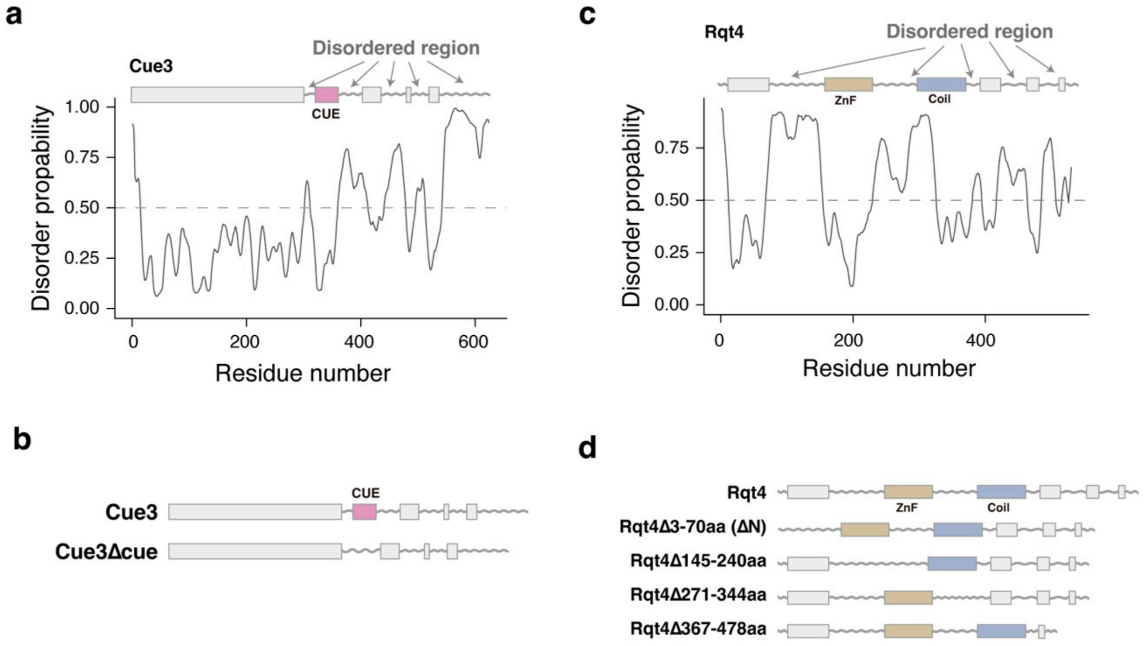

Supplementary Figure 1. Domain structure of two accessory proteins in the RQT complex

(a) Domain diagram of Cue3 and the order/disorder map along its length as predicted by PrDOS (https://prdos.hgc.jp/cgi-bin/top.cgi)[1]. **(b)** Domain diagram of Cue3 and its mutants. **(c)** Domain diagram of Rqt4 and the order/disorder map along its length as predicted by PrDOS[1]. **(d)** Domain diagram of Rqt4 and its mutants.

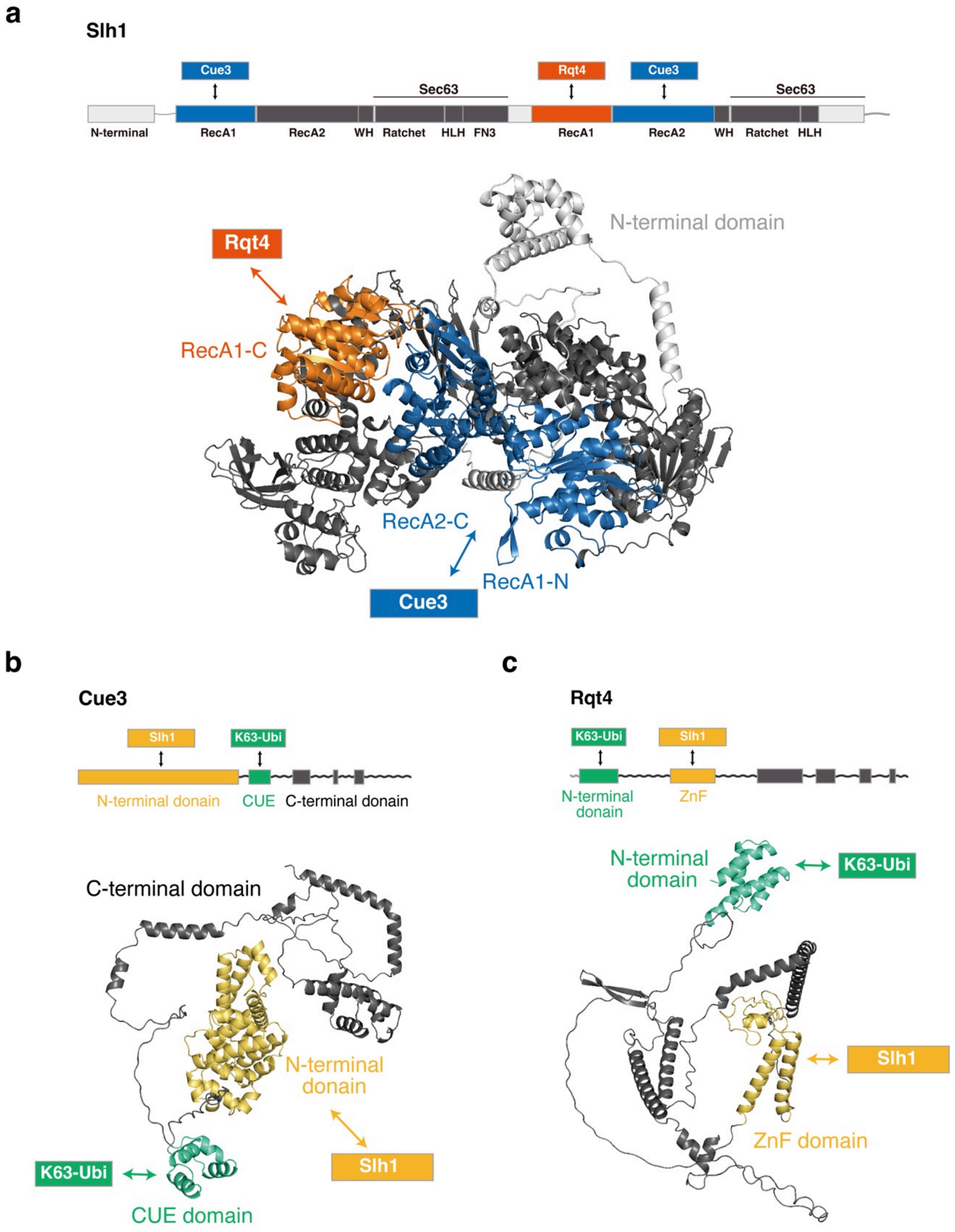

Supplementary Figure 2. Structure model of RQT components predicted by AlphaFold2

Domain diagram and 3D structure model of Slh1 **(a)**, Cue3 **(b)**, and Rqt4 **(c)** were predicted by Alphafold2[2,3]. The interacting domains among RQT factors are indicated[4].

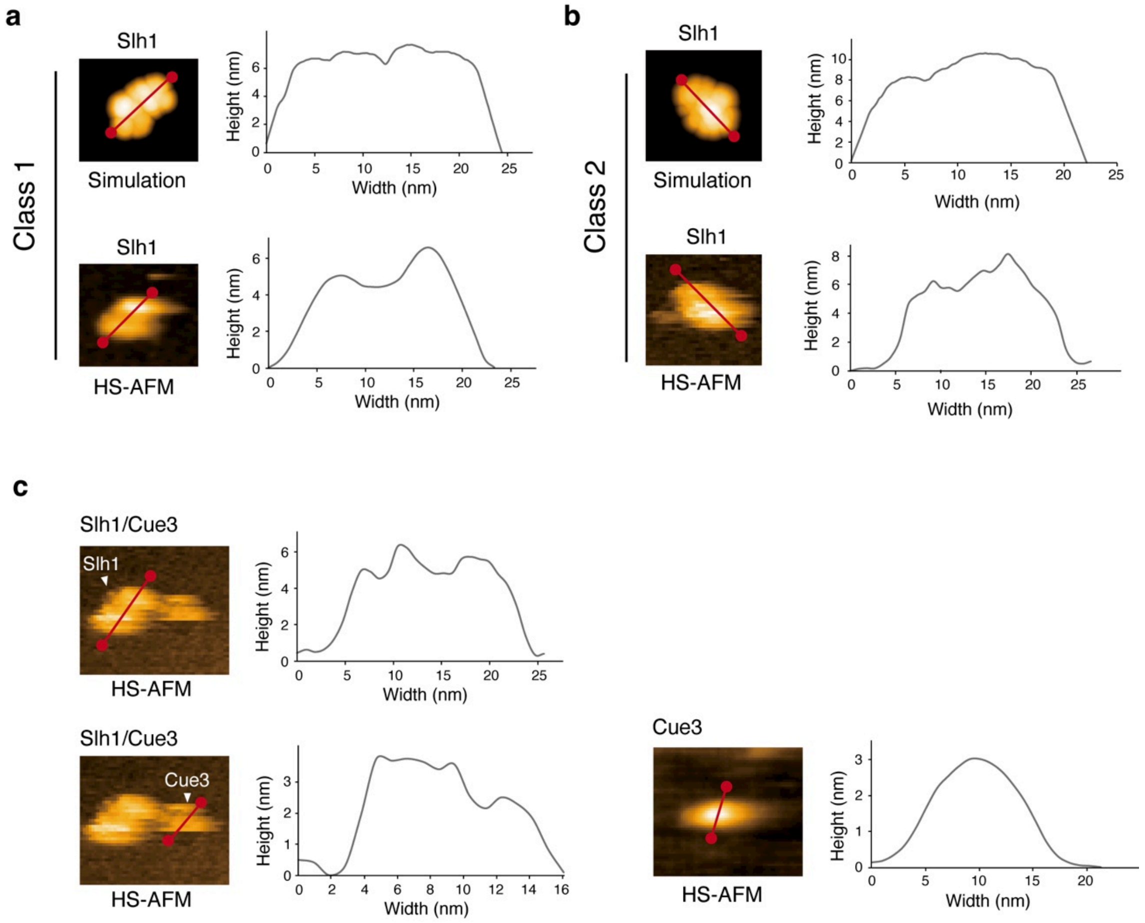

Supplementary Figure 3. Height analysis of HS-AFM images of Slh1 and Slh1/Cue3 complex
(**a**) Pseudo- and actual HS-AFM images of Slh1 belonging to Class1 particle. Line graph showing a height profile along the red line on pseudo- and actual HS-AFM images of Slh1 Class1 particle. (**b**) Pseudo- and actual HS-AFM images of Slh1 belonging to Class2 particle. Line graph showing a height profile along the red line on pseudo- and actual HS-AFM images of Slh1 Class2 particle. (**c**) HS-AFM images of Slh1/Cue3 and Cue3. Line graph showing a height profile along the red line on p HS-AFM images of Slh1/Cue3 complex and Cue3.

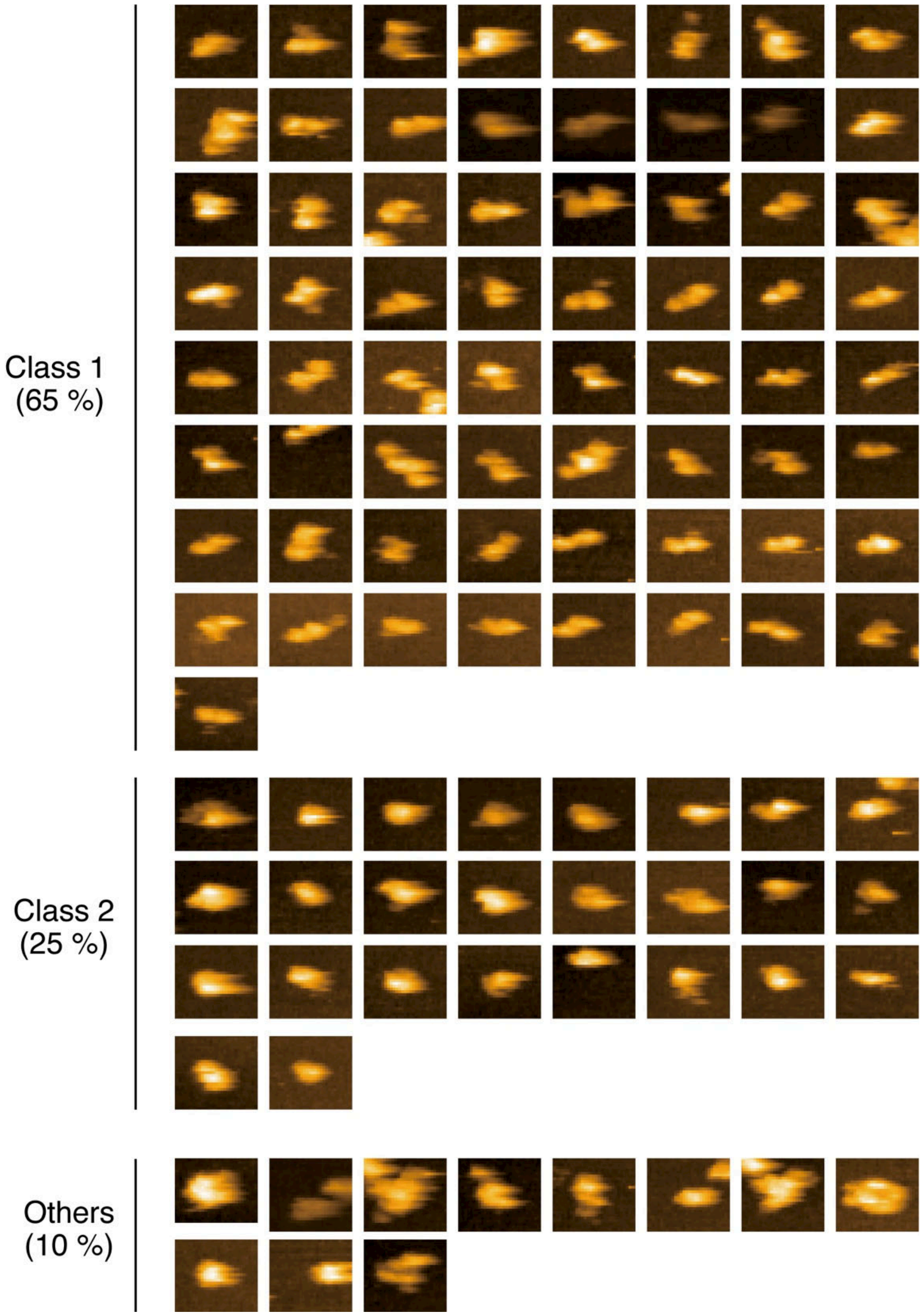

Supplementary Figure 4. HS-AFM images of Slh1 particle for Classification

The classification results in shown in Fig. 3d

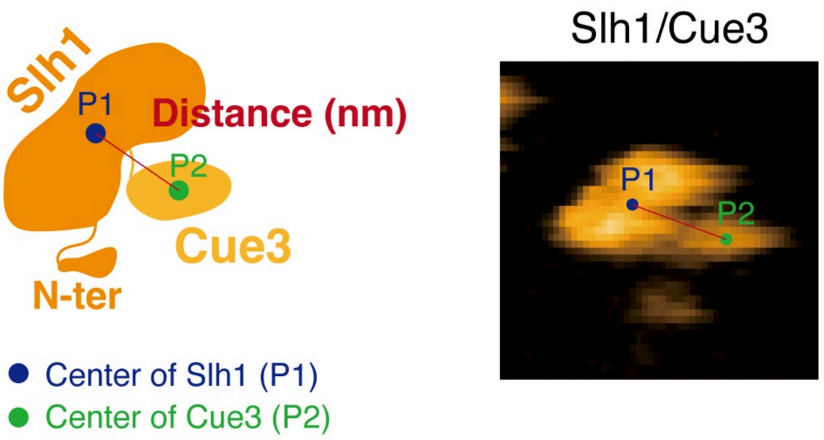

Supplementary Figure 5. The method used to obtain the distance between Slh1 and Cue3.
The center positions of Slh1 (P1) and Cue3 (P2) are determined by a tracking algorithm, and then the distance between P1 and P2 is calculated for each frame.

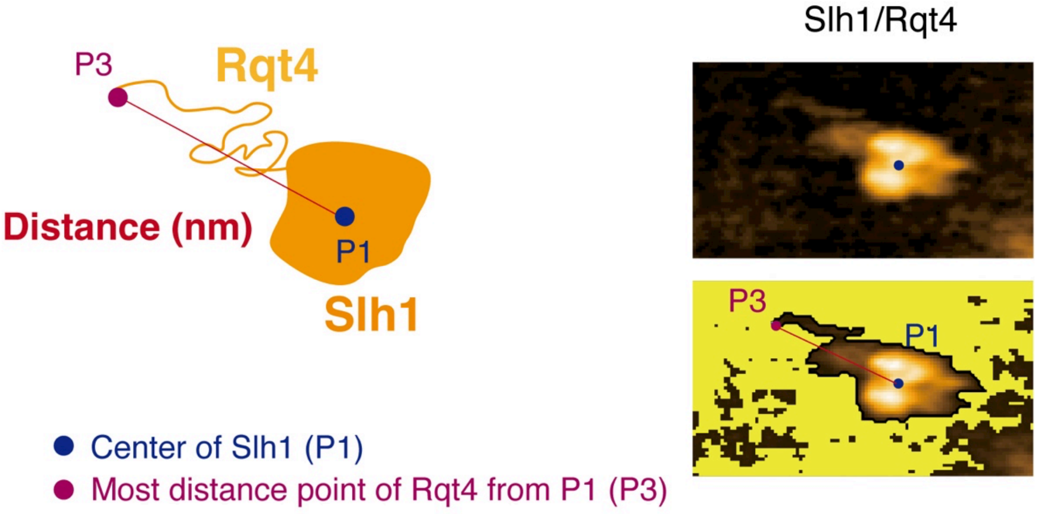

Supplementary Figure 6. The method used to obtain the distance between center of Slh1 and the most distant point of Rqt4.

The center position of Slh1 (P1) is determined by a tracking algorithm. To visualize the Rqt4, we manually set the threshold to remove the background and identify the region of Rqt4 as shown by the yellow-field region. The most distant point of Rqt4 from P1 (P3) was determined using an algorithm, and then the distance between P1 and P3 was calculated for each frame.

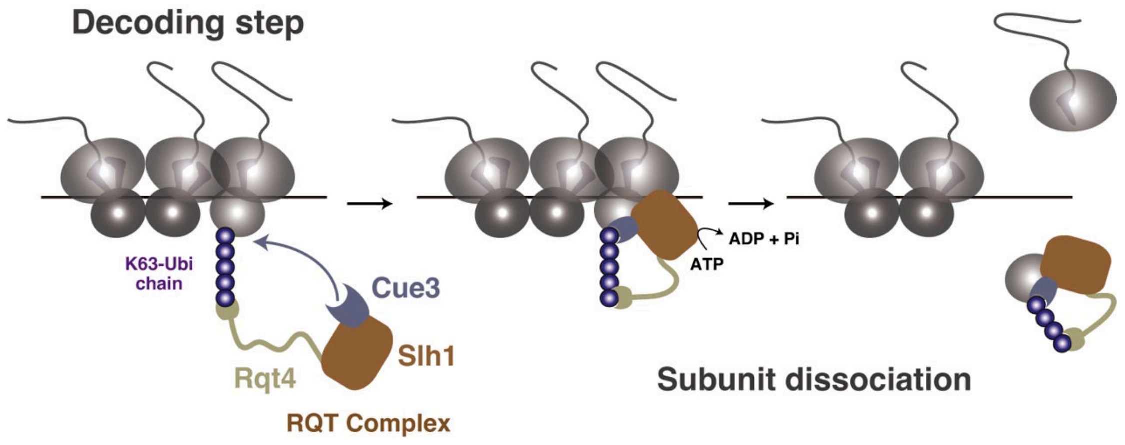

Supplementary Figure 7. Model of the decoding step of the K63-linked polyubiquitinated colliding ribosome by the RQT complex

Supplementary Table 1: The yeast strains used in this study

Name	Genotype	Source
W303a	*MATa ade2 his3 leu2 trp1 ura3 can1*	Lab.stock
ski2ΔuS10-3HA	*W303a ski2Δ::kanMX4 uS10-3HA::HISMX6*	[5]
ski2ΔuS10-3HAhel2Δ	*W303a ski2Δ::kanMX4 uS10-3HA::HISMX6 hel2Δ::natMX4*	This study
ltn1Δ	*W303a ltn1Δ::kanMX4*	[6]
ltn1Δhel2Δ	*W303a hel2Δ::natMX4 ltn1Δ::kanMX4*	[6]
ltn1ΔuS10 shuffle	*W303a us10Δ::natMX4 ltn1Δ::kanMX4*	[6]
ltn1Δslh1Δ	*W303a slh1Δ::natMX4 ltn1Δ::kanMX4*	[6]
ltn1Δcue3Δrqt4Δ	*W303a cue3::kanMX4 rqt4Δ::natMX4 ltn1Δ::hygMX4*	[6]

Supplementary Table 2: The plasmids used in this study

Name	Feature	Source
p415-Hel2-Flag	CEN, LEU2, GPD promoter, HEL2-FLAG	[7]
pGEX-Ubc4	pGEX, AmpR, tac promoter, GST-3C-UBC4	[7]
p416-Uba1-Flag	CEN, URA3, GPD promoter, UBA1-FLAG	This study
p425GAL-Slh1-FTP	2μ, LEU2, GAL1 promoter, Slh1-FTP	[5]
p425GAL-Slh1ΔN-FTP	2μ, LEU2, GAL1 promoter, Slh1ΔN-FTP	This study
p424GAL-Cue3	2μ, TRP1, GAL1 promoter, Cue3	[5]
p424GAL-Cue3-Flag	2μ, TRP1, GAL1 promoter, Cue3-Flag	This study
p424GAL-Cue3Δcue-Flag	2μ, TRP1, GAL1 promoter, Cue3ΔCUE-Flag	This study
p426GAL-Rqt4	2μ, URA3, GAL1 promoter, Rqt4	[5]
p426GAL-Rqt4-Flag	2μ, URA3, GAL1 promoter, Rqt4-Flag	This study
p426GAL-Rqt4Δ70(ΔN)-Flag	2μ, URA3, GAL1 promoter, Rqt4Δ70aa-Flag	This study
p426GAL-Rqt4Δ145-240-Flag	2μ, URA3, GAL1 promoter, Rqt4Δ145-240aa-Flag	This study
p426GAL-Rqt4Δ271-344-Flag	2μ, URA3, GAL1 promoter, Rqt4-Δ271-344aaFlag	This study
p426GAL-Rqt4Δ367-478-Flag	2μ, URA3, GAL1 promoter, Rqt4Δ367-478aa-Flag	This study
pRS415-Rqt4-Flag	CEN, LEU2, Rqt4 promoter, Rqt4-Flag	[6]
pRS415-Rqt4Δ70(ΔN)-Flag	CEN, LEU2, Rqt4 promoter, Rqt4Δ70aa-Flag	This study
pRS414-Cue3-Flag	CEN, TRP1, Cue3 promoter, Cue3-Flag	[6]
pRS414-Cue3ΔCUE-Flag	CEN, TRP1, Cue3 promoter, Cue3ΔCUE-Flag	[6]
p416GPD-HA-SDD1-V5	CEN, URA3, GPD promoter, HA-SDD1-V5	[5]

Supplementary Table 3: The oligonucleotides used in this study

Name	Sequence (5’ to 3’)
Template DNA for *SDD1* mRNA using in vitro translation	GCAAGCTAATACGACTCACTATAGGGACCAAACAAAACAAATAAAACAAAA<br>ACACAATGCATCATCATCATCATCATGAACAAAAGCTAATCTCCGAGGAAGA<br>CTTGGATCAAGTTTTAAAGTTACAAGCTACACTTTTTCCCAATGCCATACTGA<br>AACAGGTTCATCTCGACAATGCCAACATAGAAAACAAGAGAATTCTGAAGG<br>AAATTACATATAAATACCTTTCCAACGAAAACTGTAAAGAGGAGAACAAATT<br>CGGAACATTCATCGTAAAGAGAATTTTTTTTGGTGACTTATCACTCGGGGTTT<br>CCGTGTTAATCAACCGTATTGCTTTCGAATCGGCCACATCATCCATTATGGTA<br>GTGAGAAGTTCATTTATTGAAAGTGATTTCTTTTATGAAGATTATTTGATTTT<br>CGATTGCAGAGCGAAACGACGAAAAAAATAAGGATCCGAATTCCTGTCGTA<br>CACCATCAGGGTACGAGCTAGCCCATGGCGTACACCATCAGGGTACGACTAG<br>TAGAAAAAAAAAAAAAAAAAAAAAAAAAAAAAA
T7-His-SDD1-F	GCCGCAAGCTAATACGACTCACTATAGGGACCAAACAAAACAAATA<br>AAACAAAAACACAATGCATCATCATCATCATCATGAACAAAAGCTA<br>ATCTCCGAGGAAGACTTGGATCAAGTTTTAAAGTTACAAGCTAC
SDD1-213-MS2-AAA-R	TTTTTTTTTTTTTTTTTTTTTTTTTTTTTTCTACTAGTCGTACCCTGAT<br>GGTGTACGCCATGGGCTAGCTCGTACCCTGATGGTGTACGACAGGAA<br>TTCGGATCCTTATTTTTTTCGTCGTTTCGCTCT

Supplementary Table 4: The antibodies used in this study

Antibody	Company	RRID	Dilution
Anti-HA antibody	Roche	RRID: AB_390917	1:10000
Anti-Flag antibody	Sigma-Aldrich	RRID: AB_262044	1:5000
Anti-ubiquitin antibody	Santa Cruz Biotechnology	RRID: AB_628423	1:1000
Antiubiquitin (linkage-specific K48) antibody	Abcam	RRID: AB_2783797	1:1000
Antiubiquitin (linkage-specific K63) antibody	Abcam	RRID: AB_2895239	1:1000
Anti-eEF-2 antibody	Lab.stock	N/A	1:20000
Anti-mouse IgG, horseradish Peroxidase	GE Healthcare	RRID: AB_772210	1:5000
Anti-rabbit IgG, horseradish Peroxidase	GE Healthcare	RRID: AB_772206	1:5000

Supplementary References

1 Ishida, T. & Kinoshita, K. PrDOS: prediction of disordered protein regions from amino acid sequence. *Nucleic Acids Res* **35**, W460-464 (2007). https://doi.org:10.1093/nar/gkm363

2 Varadi, M. *et al.* AlphaFold Protein Structure Database: massively expanding the structural coverage of protein-sequence space with high-accuracy models. *Nucleic Acids Res* **50**, D439-d444 (2022). https://doi.org:10.1093/nar/gkab1061

3 Jumper, J. *et al.* Highly accurate protein structure prediction with AlphaFold. *Nature* **596**, 583-589 (2021). https://doi.org:10.1038/s41586-021-03819-2

4 Best, K. *et al.* Clearing of ribosome collisions by the ribosome quality control trigger complex RQT. *bioRxiv*, 2022.2004.2019.488791 (2022). https://doi.org:10.1101/2022.04.19.488791

5 Matsuo, Y. *et al.* RQT complex dissociates ribosomes collided on endogenous RQC substrate SDD1. *Nature structural & molecular biology* (2020). https://doi.org:10.1038/s41594-020-0393-9

6 Matsuo, Y. *et al.* Ubiquitination of stalled ribosome triggers ribosome-associated quality control. *Nature communications* **8**, 159 (2017). https://doi.org:10.1038/s41467-017-00188-1

7 Ikeuchi, K. *et al.* Collided ribosomes form a unique structural interface to induce Hel2-driven quality control pathways. *The EMBO journal* (2019). https://doi.org:10.15252/embj.2018100276
