# Supplementary material for: Decoding of the ubiquitin code for clearance of colliding ribosomes by the RQT complex
Source: Nat Commun. 2023 Jan 10;14:79. doi: 10.1038/s41467-022-35608-4 (PMC9831982; doi:10.1038/s41467-022-35608-4)
Supplement: Supplementary file 2 — Description of Additional Supplementary Files [file 41467_2022_35608_MOESM2_ESM.pdf]

Description of Additional Supplementary Files

Supplementary Movie 1: HS-AFM movie of Slh1 Class1 particle

Supplementary Movie 2: HS-AFM movie of Slh1 Class2 particle

Supplementary Movie 3: HS-AFM movie of Slh1ΔN

Supplementary Movie 4: HS-AFM movie of Cue3

Supplementary Movie 5: HS-AFM movie of Rq4

Supplementary Movie 6: HS-AFM movie of Slh1/Cue3 complex

Supplementary Movie 7: HS-AFM movie of Slh1/Rqt4 complex

Supplementary Movie 8: HS-AFM movie of Slh1/Cue3/Rqt4 complex
