# Supplementary material for: Decoding of the ubiquitin code for clearance of colliding ribosomes by the RQT complex
Source: Nat Commun. 2023 Jan 10;14:79. doi: 10.1038/s41467-022-35608-4 (PMC9831982; doi:10.1038/s41467-022-35608-4)
Supplement: Supplementary file 11 — Reporting Summary [file 41467_2022_35608_MOESM11_ESM.pdf]

nature portfolio

Corresponding author(s): Yoshitaka Matsuo

Last updated by author(s): 2022/11/30

Reporting Summary

Nature Portfolio wishes to improve the reproducibility of the work that we publish. This form provides structure for consistency and transparency in reporting. For further information on Nature Portfolio policies, see our Editorial Policies and the Editorial Policy Checklist.

Statistics

For all statistical analyses, confirm that the following items are present in the figure legend, table legend, main text, or Methods section.

n/a	Confirmed	
☐	☒	The exact sample size (*n*) for each experimental group/condition, given as a discrete number and unit of measurement
☐	☒	A statement on whether measurements were taken from distinct samples or whether the same sample was measured repeatedly
☒	☐	The statistical test(s) used AND whether they are one- or two-sided<br>*Only common tests should be described solely by name; describe more complex techniques in the Methods section.*
☒	☐	A description of all covariates tested
☒	☐	A description of any assumptions or corrections, such as tests of normality and adjustment for multiple comparisons
☒	☐	A full description of the statistical parameters including central tendency (e.g. means) or other basic estimates (e.g. regression coefficient) AND variation (e.g. standard deviation) or associated estimates of uncertainty (e.g. confidence intervals)
☒	☐	For null hypothesis testing, the test statistic (e.g. *F*, *t*, *r*) with confidence intervals, effect sizes, degrees of freedom and *P* value noted<br>*Give P values as exact values whenever suitable.*
☒	☐	For Bayesian analysis, information on the choice of priors and Markov chain Monte Carlo settings
☒	☐	For hierarchical and complex designs, identification of the appropriate level for tests and full reporting of outcomes
☒	☐	Estimates of effect sizes (e.g. Cohen's *d*, Pearson's *r*), indicating how they were calculated

Our web collection on statistics for biologists contains articles on many of the points above.

Software and code

Policy information about availability of computer code

Data collection	Eagle software package 1.0.2.7 by RIBM in an Igor Pro-6 (WaveMetrics: ver 6.37)
Data analysis	FalconViewer based on Igor Pro-9 (WaveMetrics: ver 9.01), AlphaFold2

For manuscripts utilizing custom algorithms or software that are central to the research but not yet described in published literature, software must be made available to editors and reviewers. We strongly encourage code deposition in a community repository (e.g. GitHub). See the Nature Portfolio guidelines for submitting code & software for further information.

Data

Policy information about availability of data

All manuscripts must include a data availability statement. This statement should provide the following information, where applicable:

- Accession codes, unique identifiers, or web links for publicly available datasets
- A description of any restrictions on data availability
- For clinical datasets or third party data, please ensure that the statement adheres to our policy

Any data relating to the findings presented in this article are available within the article and its supplementary information files. All source data are provided with this paper as Source data file.

Human research participants

Policy information about studies involving human research participants and Sex and Gender in Research.

Reporting on sex and gender	Not applicable. Because no human research in this study
Population characteristics	Not applicable. Because no human research in this study
Recruitment	Not applicable. Because no human research in this study
Ethics oversight	Not applicable. Because no human research in this study

Note that full information on the approval of the study protocol must also be provided in the manuscript.

Field-specific reporting

Please select the one below that is the best fit for your research. If you are not sure, read the appropriate sections before making your selection.

☒ Life sciences ☐ Behavioural & social sciences ☐ Ecological, evolutionary & environmental sciences

For a reference copy of the document with all sections, see nature.com/documents/nr-reporting-summary-flat.pdf

Life sciences study design

All studies must disclose on these points even when the disclosure is negative.

Sample size	No statistical methods were used to predetermine the sample size. Number of the particle for the classification of Slh1 particle by HS-AFM (Figure 3d and supplementary figure 4) was chosen based on our preliminary experiments; more than 100 particles were established as sufficient to classify the major two different orientation of the particles on the mica surface.
Data exclusions	No data exclusions
Replication	The experiments were performed at least twice with consistent results.
Randomization	No randomrization. Our study compared proteins dynamics by HS-AFM, and protein function analyzed in vitro and in vivo using yeast clones in which different genes were deleted or modified in parallel.
Blinding	The investigators were not blinded during data collection.

Reporting for specific materials, systems and methods

We require information from authors about some types of materials, experimental systems and methods used in many studies. Here, indicate whether each material, system or method listed is relevant to your study. If you are not sure if a list item applies to your research, read the appropriate section before selecting a response.

Materials & experimental systems

n/a	Involved in the study	
☐	☒	Antibodies
☒	☐	Eukaryotic cell lines
☒	☐	Palaeontology and archaeology
☒	☐	Animals and other organisms
☒	☐	Clinical data
☒	☐	Dual use research of concern

Methods

n/a	Involved in the study	
☒	☐	ChIP-seq
☒	☐	Flow cytometry
☒	☐	MRI-based neuroimaging

Antibodies

Antibodies used	The details of all antibodies used in this study were described in the supplementary table 4.<br><br>Anti-HA antibody (Roche, RRID: AB_390917, Cat#: 12013819001, 1:10000 dilution)<br>Anti-Flag antibody (Sigma-Aldrich, RRID: AB_262044, Cat#: F1804-1MG, 1:5000 dilution)<br>Anti-ubiquitin antibody (Santa Cruz Biotechnology, RRID: AB_628423, Cat#: sc-8017, 1:1000 dilution)

Antiubiquitin (linkage-specific K48) antibody (Abcam, RRID: AB_2783797, Cat# ab140601, 1:1000 dilution)
Antiubiquitin (linkage-specific K63) antibody (Abcam, RRID: AB_2895239, Cat# ab179434, 1:1000 dilution)
Anti-eEF-2 antibody (Lab.stock, N/A, 1:20000 dilution)
Anti-mouse IgG, horseradish Peroxidase (Cytiva, RRID:AB_772210, Cat# NA931-1ML, 1:5000 dilution)
Anti-rabbit IgG, horseradish Peroxidase (Cytiva, RRID:AB_772206, Cat# NA934-1ML, 1:5000 dilution)

Validation

The validations of all antibodies refer to as follow,
Anti-HA antibody (https://www.sigmaaldrich.com/JP/ja/product/roche/12013819001)
Anti-Flag antibody (https://www.sigmaaldrich.com/JP/ja/product/sigma/f1804)
Anti-ubiquitin antibody (https://datasheets.scbt.com/sc-8017.pdf)
Antiubiquitin (https://www.abcam.co.jp/ubiquitin-linkage-specific-k48-antibody-ep8589-ab140601.html)
Antiubiquitin (https://www.abcam.co.jp/ubiquitin-linkage-specific-k63-antibody-epr8590-448-ab179434.html)
Anti-eEF-2 antibody (Ikeuchi et al., 2019 EMBO J: PMID: 30609991)
Anti-mouse IgG, horseradish Peroxidase (https://www.sigmaaldrich.com/JP/ja/product/sigma/gena9311ml)
Anti-rabbit IgG, horseradish Peroxidase (https://www.sigmaaldrich.com/JP/ja/product/sigma/gena9341ml)
