# Supplementary material for: Decoding of the ubiquitin code for clearance of colliding ribosomes by the RQT complex
Source: Nat Commun. 2023 Jan 10;14:79. doi: 10.1038/s41467-022-35608-4 (PMC9831982; doi:10.1038/s41467-022-35608-4)
Supplement: Supplementary file 12 — Peer review report [file 41467_2022_35608_MOESM12_ESM.pdf]

nature portfolio

Peer Review File

Decoding of the ubiquitin code for clearance of colliding ribosomes by the RQT complex

Open Access This file is licensed under a Creative Commons Attribution 4.0 International License, which permits use, sharing, adaptation, distribution and reproduction in any medium or format, as long as you give appropriate credit to the original author(s) and the source, provide a link to the Creative Commons license, and indicate if changes were made. In the cases where the authors are anonymous, such as is the case for the reports of anonymous peer reviewers, author attribution should be to 'Anonymous Referee' followed by a clear attribution to the source work. The images or other third party material in this file are included in the article's Creative Commons license, unless indicated otherwise in a credit line to the material. If material is not included in the article's Creative Commons license and your intended use is not permitted by statutory regulation or exceeds the permitted use, you will need to obtain permission directly from the copyright holder. To view a copy of this license, visit http://creativecommons.org/licenses/by/4.0/.

REVIEWER COMMENTS

Reviewer #1 (Remarks to the Author):

During RQC, Hel2 ubiquitinates uS10 of the stalled ribosome, which is recognized and split into individual subunits by the RQT complex. The RQT is composed of the helicase Slh1, the ubiquitin-binding protein Cue3 (Rqt3) and a zinc finger protein Rqt4. In previous work from the Inada group (Matsuo et al Nat Comm 2017), deletion of Cue3 did not completely suppress RQC activity, suggesting that an additional factor recognizes the ubiquitinated uS10 – a prime candidate was Rqt4 since double deletion of Rqct4 and Cue3 was sufficient to completely suppress RQC activity. Here the authors confirm this assumption showing that CUE domain of Cue3 and the N-terminal domain of Rqc4 bind to K63-linked ubiquitin chain independently of each other.
The experiments are well-performed and clearly presented. The manuscript is well-written and easy to follow. The conclusions are supported by the data. Overall, the findings provide a relatively small increment in the mechanism by which RQT operates, which will be of interest mainly to those working directly in the field. It is a shame that the involvement of Rqt4 in recognizing other ubiquitin-codes of ribosomes was not undertaken, for example, in the 18S non-functional rRNA decay pathway, which would provide more interest to a wider audience.

Another small point.
In the 2017 paper, the authors rename Cue3 as Rqt3 but in this paper, Cue3 is used throughout.

Reviewer #2 (Remarks to the Author):

Matsuo and Inada explore the role of Cue3 and Rqt4 in splitting of collided ribosomes. They use in vitro and in vivo assays and structural analysis to conclude that Cue3 and Rqt4 recognize K63 ubiquitin on stalled ribosomes, allowing the RQT to target stalled ribosomes and split them. The most interesting finding is that Cue3 has a short range of ubiquitin detection and Rqt4 has a longer range, leading the authors to speculate that Rqt4 allows crosstalk between the RQT and the NRD pathway, so that in some cases stalled ribosomes are destroyed rather than recycled. However this connection is not explicitly explored in this work. This is a valuable mechanistic study (while somewhat incremental), and addresses the question of why there are two seemingly redundant members of the RQT complex that when deleted cause only partial defects. Understanding the mechanism of RQT and RQC in general is of high importance. I recommend publication.

Reviewer #3 (Remarks to the Author):

Matsuo and Inaba investigated the first step of the RQT system in rescuing ribosome collisions, the fundamental biological question of how the RQT complex recognizes ubiquitin chains by biochemical methods and direct visualization by HS-AFM. By taking advantage of the capability of HS-AFM, the authors were able to visualize the intrinsically disordered regions in the RQT complex and they propose that flexible IDRs are essential to recognize ubiquitin chains for the RQT system based on the HS-AFM observations. The proposed model is intriguing, and the effective use of the new technique of HS-AFM is commendable; thus, the reviewer considers the study is fundamentally worthy of publication. On the other hand, from the viewpoint of the reviewer, who is an AFM expert, the interpretation of the AFM data shown in this manuscript seems to be too naive and lacking in rigor. Since the HS-AFM data is crucial to the conclusions drawn in this study, the interpretation should be more robust, and the analysis should be improved to be more convincing.
The most serious flaw of the AFM data in this manuscript is the validity of the protein assignments to the particles seen in the AFM image. The most easily convincing state in the AFM image is one in which all particles have the same shape, but their orientation is random. On the other hand, although particles of various shapes can be seen in the images shown in the manuscript, the authors assign specific shapes of particles to the component proteins of the RQT complex almost without deep consideration. Solid data should be presented on what the AFM image corresponds to. Below, I point

out the problems and suggest improvements for each data set.

Fig. 3A: The authors assign several differently shaped particles in the AFM image as different orientations of Slh1. Usually, during AFM observations, proteins are in contact with the substrate in most favorite orientation based on some physical properties such as maximum ground area and charge distribution, etc..., so there is not a lot of shape diversity if protein conformation is homogeneous. In this sense, the images shown by the authors have quite a variety of forms and a questionable level of purity. Even if it is challenging to measure particles with precisely the same shape, the authors should at least classify the shape from the AFM images, even if by eye inspection, and discuss how much diversity in the observed shape. Then, for the highest number of particles, a pseudo-AFM image should be obtained from the putative structure by collision simulation and compared in shape and height with the actual image before drawing conclusions. The AFM image should also have a Z color bar to indicate height information.
The authors conclude that the region fluctuating in the AFM image corresponds to the N-terminal region of Slh1, but to strictly exclude the possibility that it is some other region, the protein without the N-terminal region should be observed.

Fig. 3B: As in Fig. 3A, there is no comparison between the shape classification and the pseudo-AFM image, so it cannot be determined whether the particles in the AFM image correspond to the Slh1-Cue3 complex.

Figs. 3B-D: AFM images of complexes of different combinations of Slh1, Cue3, and Rqt4 are shown, but there are no images of each alone, so it is unclear if the image assignments are valid. AFM images of each protein alone should also be described.

Fig. 3E: I In this analysis, the size of the bright spots, which the authors consider as Cue3, is measured, but this measurement is, in general, not appropriate for the analysis of AFM images. The width of the bright spots is inherently ambiguous and should be strictly defined as the width at half maximum of the peak from the cross-sectional view. In addition, the convolution effect of probe size makes the width of the spots ambiguous since it varies with the probe-end condition. Rather, the distance between the peaks of the two bright spots (in this case, the two blobs, Cue and Slh1) should be measured. This way, the object being measured can be defined more precisely, and what the authors want to discuss, i.e., the movable range of Cue3, can be discussed with more solid values.

Fig. 3F: This analysis is also rather vague and lacks rigor. How did the authors determine the two ends from a rather blurred image? I assume that the author probably measured by visual inspection. The conclusion itself is not likely to change, but as long as the authors discuss quantitatively, the values should be extracted by analysis based on objective indices. The analysis used in the following paper, for example, might be useful.
https://pubs.rsc.org/en/content/articlelanding/2020/cc/d0cc03776a

Supplementary Movies: Since there is no information about imaging speed (frame time) for all movies, it is impossible to know if these movies are played at real-time speed. To know the time scale of the fluctuation of the IDR, the image acquisition time should be described in the movie caption. If the movie is played in not real-time, it should also indicate how many times faster it is played. The author may also indicate the elapsed time in the movies.

Supplementary Movie 2: The authors state from this movie that Cue3 is moved around Slh1, but I am not sure which part of the movie they are referring to. The authors should indicate Cue3 with an arrow or something in the movie.

Response to Reviewers

We thank all reviewers for their positive, helpful, and insightful comments. We are happy to provide the additional data requested by the reviewer 3 that has strengthened the manuscript. In our detailed response, the reviewers' comments are Italicized whereas our response is in Roman typeface with blue color.

REVIEWER COMMENTS

Reviewer #1 (Remarks to the Author):
During RQC, Hel2 ubiquitinates uS10 of the stalled ribosome, which is recognized and split into individual subunits by the RQT complex. The RQT is composed of the helicase Slh1, the ubiquitin-binding protein Cue3 (Rqt3) and a zinc finger protein Rqt4. In previous work from the Inada group (Matsuo et al Nat Comm 2017), deletion of Cue3 did not completely suppress RQC activity, suggesting that an additional factor recognizes the ubiquitinated uS10 – a prime candidate was Rqt4 since double deletion of Rqct4 and Cue3 was sufficient to completely suppress RQC activity. Here the authors confirm this assumption showing that CUE domain of Cue3 and the N-terminal domain of Rqc4 bind to K63-linked ubiquitin chain independently of each other.
The experiments are well-performed and clearly presented. The manuscript is well-written and easy to follow. The conclusions are supported by the data. Overall, the findings provide a relatively small increment in the mechanism by which RQT operates, which will be of interest mainly to those working directly in the field. It is a shame that the involvement of Rqt4 in recognizing other ubiquitin-codes of ribosomes was not undertaken, for example, in the 18S non-functional rRNA decay pathway, which would provide more interest to a wider audience.

We appreciate the positive feedback by the reviewer. We will attempt to address the involvement of Rqt4 in recognizing other ubiquitin codes of ribosomes including the 18S NRD in future work.

Another small point.
In the 2017 paper, the authors rename Cue3 as Rqt3 but in this paper, Cue3 is used throughout.

We thank the reviewer for his/her comments. Cue3 is still a common name in the field after we renamed Cue3 as Rqt3 in the 2017 paper, so we use Cue3 here.

Reviewer #2 (Remarks to the Author):
Matsuo and Inada explore the role of Cue3 and Rqt4 in splitting of collided ribosomes. They use in vitro and in vivo assays and structural analysis to conclude that Cue3 and Rqt4 recognize K63 ubiquitin on stalled ribosomes, allowing the RQT to target stalled ribosomes and split them. The most interesting finding is that Cue3 has a short range of ubiquitin detection and Rqt4 has a longer range, leading the authors to speculate that Rqt4 allows crosstalk between the RQT and the NRD pathway, so that in some cases stalled ribosomes are destroyed rather than recycled. However this connection is not explicitly explored in this work. This is a valuable mechanistic study (while somewhat incremental), and addresses the question of why there are two seemingly redundant members of the RQT complex that when deleted cause only partial defects. Understanding the mechanism of RQT and RQC in general is of high importance. I recommend publication.

We thank the reviewer for the excellent evaluation of our work.

Reviewer #3 (Remarks to the Author):

Matsuo and Inaba investigated the first step of the RQT system in rescuing ribosome collisions, the fundamental biological question of how the RQT complex recognizes ubiquitin chains by biochemical methods and direct visualization by HS-AFM. By taking advantage of the capability of HS-AFM, the authors were able to visualize the intrinsically disordered regions in the RQT complex and they propose that flexible IDRs are essential to recognize ubiquitin chains for the RQT system based on the HS-AFM observations. The proposed model is intriguing, and the effective use of the new technique of HS-AFM is commendable; thus, the reviewer considers the study is fundamentally worthy of publication. On the other hand, from the viewpoint of the reviewer, who is an AFM expert, the interpretation of the AFM data shown in this manuscript seems to be too naive and lacking in rigor. Since the HS-AFM data is crucial to the conclusions drawn in this study, the interpretation should be more robust, and the analysis should be improved to be more convincing.

The most serious flaw of the AFM data in this manuscript is the validity of the protein assignments to the particles seen in the AFM image. The most easily convincing state in the AFM image is one in which all particles have the same shape, but their orientation is random. On the other hand, although particles of various shapes can be seen in the images shown in the manuscript, the authors assign specific shapes of particles to the component proteins of the RQT complex almost without deep consideration. Solid data should be presented on what the AFM image corresponds to. Below, I point out the problems and suggest improvements for each data set.

We thank the reviewer very much for the helpful and insightful comments, which greatly contribute to improving our manuscript.

Fig. 3A: The authors assign several differently shaped particles in the AFM image as different orientations of Slh1. Usually, during AFM observations, proteins are in contact with the substrate in most favorite orientation based on some physical properties such as maximum ground area and charge distribution, etc..., so there is not a lot of shape diversity if protein conformation is homogeneous. In this sense, the images shown by the authors have quite a variety of forms and a questionable level of purity. Even if it is challenging to measure particles with precisely the same shape, the authors should at least classify the shape from the AFM images, even if by eye inspection, and discuss how much diversity in the observed shape. Then, for the highest number of particles, a pseudo-AFM image should be obtained from the putative structure by collision simulation and compared in shape and height with the actual image before drawing conclusions. The AFM image should also have a Z color bar to indicate height information.

We apologize for raising doubt about the purity of the sample by picking the wrong HS-AFM image of Slh1 for the previous Fig. 3A. To address the reviewer’s concerns, we reanalyzed the Slh1 by HS-AFM and classified their shapes (Revised Supplementary Figure 4). As suggested by the reviewer, we could find two main classes, not a lot of shape diversity; 65 % and 25 % of the Slh1 particles belong to Class1 and Class2, respectively (Revised Figure 3a-d). We next generated the pseudo-AFM images of Class1- and Class2-Slh1 particles from the Alphafold2 predicted structure lacking a flexible N-terminal region (Revised Figure 3b). This showed that the simulated-AFM images displayed similar shapes and heights compared to the actual AFM images of Slh1 (Figure 3c and revised Supplementary Figure 3a-b). So, we concluded that these particles are Slh1. These results let us focus on Class1 particles for other HS-AFM analyses.

We have described these points in the revised manuscript and added the Z color bar in all HS-AFM images in the revised figures.

The authors conclude that the region fluctuating in the AFM image corresponds to the N-terminal region of Slh1, but to strictly exclude the possibility that it is some other region, the protein without the N-terminal region should be observed.

To verify the reviewer's concerns, we constructed the Slh1 mutant lacking an N-terminal region (3-217aa: Slh1ΔN) and analyzed it by HS-AFM. As expected, the fluctuating region of Slh1 (Supplementary Movie 1) completely disappeared in the HS-AFM image and movie of Slh1ΔN (revised Figure 3e and Supplementary Movie 3). This indicated that the fluctuating region of Slh1 is indeed an N-terminal region. We have described this in the revised manuscript.

Fig. 3B: As in Fig. 3A, there is no comparison between the shape classification and the pseudo-AFM image, so it cannot be determined whether the particles in the AFM image correspond to the Slh1-Cue3 complex.

We have classified the shapes of Slh1 and found the particle of Class1 as a major particle in Slh1 (Revised supplementary figure 3). So, we here focused on Class1 particles with Cue3. We compared the shape of Slh1(Revised figure 3c and supplementary Movie 1) and Slh1/Cue3 heterodimer (Revised figure 3i and supplementary Movie 6) belonging to Class1 particles, which clearly showed that the additional barrel-shaped molecule was associated with Slh1 (Revised figure 3i and supplementary Movie 6). Furthermore, the height of this molecule is consistent with Cue3 (revised figure 3f and supplementary figure 3c), concluding that the associated barrel-shaped molecule is Cue3. We have described these points in the revised manuscript.
Since Cue3 has a lot of IDRs (Supplementary figures 1a and 2b), we cannot simulate the precise HS-AFM image of full-length Cue3 using the Cue3 PDB file predicted by Alphafold2.

Figs. 3B-D: AFM images of complexes of different combinations of Slh1, Cue3, and Rqt4 are shown, but there are no images of each alone, so it is unclear if the image assignments are valid. AFM images of each protein alone should also be described.

As advised by the reviewer, we analyzed a single molecule of Cue3 and Rqt4 by HS-AFM, which are described in the revised figures 3f and 3g. Cue3 looked smaller than Slh1 and its height was around 3 nm (Revised figure 3f, supplementary figure 4c, and supplementary Movie 4). Rqt4 looked like a string protein (Revised figure 3g and supplementary Movie 5), which can be observed in the Slh1/Rqt4 complex (Revised figure 3j-k). These results help us with the assignment of each protein in the RQT complex (Revised figure 3i and supplementary Movie 8). We have described these points in the revised manuscript.

Fig. 3E: In this analysis, the size of the bright spots, which the authors consider as Cue3, is measured, but this measurement is, in general, not appropriate for the analysis of AFM images. The width of the bright spots is inherently ambiguous and should be strictly defined as the width at half maximum of the peak from the cross-sectional view. In addition, the convolution effect of probe size makes the width of the spots ambiguous since it varies with the probe-end condition. Rather, the distance between the peaks of the two bright spots (in this case, the two blobs, Cue and Slh1) should be measured. This way, the object being measured can be defined more precisely, and what the authors want to discuss, i.e., the movable range of Cue3, can be discussed with more solid values.

According to the reviewer's suggestion, we independently determined the center positions of Slh1 (P1) and Cue3 (P2) using a tracking algorithm, and then the distance between P1 and P2 was calculated for each frame as described in the revised supplementary figure 5. These values were plotted in the scatter plot (revised figure 4b) and histogram (revised figure 4e). Using these solid values, we discussed the movable range of Cue3 in the revised manuscripts.

Fig. 3F: This analysis is also rather vague and lacks rigor. How did the authors determine the two ends from a rather blurred image? I assume that the author probably measured by visual inspection. The conclusion itself is not likely to change, but as long as the authors discuss quantitatively, the values should be extracted by analysis based on objective indices. The analysis used in the following paper, for example, might be useful.
https://pubs.rsc.org/en/content/articlelanding/2020/cc/d0cc03776a

According to the reviewer's comments, we defined two points as described in the revised supplementary figure 6 to measure the distance between the center of Slh1 (P1) and the most distant point of Rqt4 (P3) from P1. We first determined the center position of Slh1 (P1) using the same method commented on in the previous response. To visualize the Rqt4, we manually set the threshold to remove the background and identify the region of Rqt4 as shown by the yellow-field region in the revised supplementary figure 6. The most distant point of Rqt4 from P1 (P3) was determined using an algorithm, and then the distance between P1 and P3 was calculated for each frame. The values were plotted in the scatter plot (revised figure 4d) and histogram (revised figure 4e). Using these solid values, we discussed the movable range of Rqt4 in the revised manuscripts.

Supplementary Movies: Since there is no information about imaging speed (frame time) for all movies, it is impossible to know if these movies are played at real-time speed. To know the time scale of the fluctuation of the IDR, the image acquisition time should be described in the movie caption. If the movie is played in not real-time, it should also indicate how many times faster it is played. The author may also indicate the elapsed time in the movies.

We apologize for missing the information about imaging speed. We have revised all movies, which monitored only a single molecule (Supplementary Movie 1-8). According to the reviewer's comments, we added frame rate, scall bar, and elapsed time in all movies.

Supplementary Movie 2: The authors state from this movie that Cue3 is moved around Slh1, but I am not sure which part of the movie they are referring to. The authors should indicate Cue3 with an arrow or something in the movie.

We revised the movies, which are focused on the single Slh1 particle of Class1 with or without accessory proteins (Cue3 and Rqt4). According to the reviewer’s comments, we added the caption with an arrow for each molecule including the fluctuated region in all movies.

REVIEWERS' COMMENTS

Reviewer #4 (Remarks to the Author):

The reviewer considers that the study is worthy of publication. The biological question addressed concerning the mechanisms of recognition of ubiquitin by the RQT complex is significant and of broad impact. The authors have well addressed the flaws identified in the previous version of the manuscript, in particular, those concerning the domain of expertise of the reviewer, the afm. Yet, a small number of issues remain that are detailed next:

Major Issue

1) In the new version, the authors have classified the hs-afm videos of the RQT complex into two subsets; each one corresponds to an orientation of the molecule on the mica substrate, these were termed Class1 and Class2. In the case of Class1, the authors mention: ' In Class1 particles, two globular domains, which are consistent with two RecA-like helicase domains, were observed'. In the case of Class2, the authors point out that the two globular domains are not observed. The authors perform a count and find that in most of their videos the RQT complex is oriented in Class1, out of the total, the population of Class1 is 65% and of Class2 is 25%. Subsequently, the authors restrain their analysis to the RQT complexes in the Class1 orientation. The authors justify the choice of limiting their analysis to the Class1 orientation because Class1 is more abundant.

The reviewer does not agree with the criteria of selection of the subset of data Class1: It is not because some conformation is slightly less frequent that it is less relevant from a scientific perspective: A minority of events can be as critical, or more, for scientific knowledge than the most abundant ones. Therefore, the authors must modify the justification of the selection criterium of the subgroup of Class1 orientations or expand their analysis to both subgroups Class1 and Class2 (even if the extent of the analysis of Class2 can be smaller than that of Class1 or incorporated in the Supplementary Material). Additionally, the reviewer suggests to the authors that a criterion like the following: 'the orientation of Class1 provides better access to the AFM tip to the imaging of the appropriate zones of proteins for the analysis', or similar, should be used instead of that of 'the most abundant population'.

Minor Issues

1) The authors must detail how they generated the pseudo-AFM images from the alphafold results and the possible software packages they utilized

2) Line 226. The authors write that Cue3 'engages the RQT complex at the right position', the term 'right position' is vague. The authors should clarify to the reader the meaning of 'right position'.

3) The authors show in their hs-afm movies that the Rqt4 domain explores the area surrounding the Slh1. This finding is key for the model the authors propose. For the analysis of the motion of the Rqt4 domain, the authors correctly measure the distance between the center of mass of the Slh1 and the end of the Rqt4. It is found that the Rqt4 explores a radial distance that reaches 20-40 nanometres. Nevertheless, the authors do not provide any information on the angular positions that the Rqt4 explores; it is not clarified whether the Rqt4 explores a certain range of angles with respect Slh1 in preference or not, such information would be of interest for the assessment of the probabilities of binding of Rqt4 to the K63-Ubi chain. This information could be shown in the form of radial plots that would substitute the ones of Fig.4b and Fig.4d, and/or in the form of an image of the zone of exploration that would be obtained using for instance the maximum filter for stacks available in the ImageJ; please find below an sample of a modified Fig.4 showing the maximum filter images of the movies 6 and 7 that are titled 'Maximum height from all frames'.

Response to Reviewers

We thank reviewer 4 for his/her positive, helpful, and insightful comments. In our detailed response, the reviewers' comments are Italicized whereas our response is in Roman typeface with blue color.

REVIEWERS' COMMENTS

Reviewer #4 (Remarks to the Author):

The reviewer considers that the study is worthy of publication. The biological question addressed concerning the mechanisms of recognition of ubiquitin by the RQT complex is significant and of broad impact. The authors have well addressed the flaws identified in the previous version of the manuscript, in particular, those concerning the domain of expertise of the reviewer, the afm. Yet, a small number of issues remain that are detailed next:

Major Issue

1) In the new version, the authors have classified the hs-afm videos of the RQT complex into two subsets; each one corresponds to an orientation of the molecule on the mica substrate, these were termed Class1 and Class2. In the case of Class1, the authors mention: ' In Class1 particles, two globular domains, which are consistent with two RecA-like helicase domains, were observed'. In the case of Class2, the authors point out that the two globular domains are not observed. The authors perform a count and find that in most of their videos the RQT complex is oriented in Class1, out of the total, the population of Class1 is 65% and of Class2 is 25%. Subsequently, the authors restrain their analysis to the RQT complexes in the Class1 orientation. The authors justify the choice of limiting their analysis to the Class1 orientation because Class1 is more abundant.

The reviewer does not agree with the criteria of selection of the subset of data Class1: It is not because some conformation is slightly less frequent that it is less relevant from a scientific perspective: A minority of events can be as critical, or more, for scientific knowledge than the most abundant ones. Therefore, the authors must modify the justification of the selection criterium of the subgroup of Class1 orientations or expand their analysis to both subgroups Class1 and Class2 (even if the extent of the analysis of Class2 can be smaller than that of Class1 or incorporated in the Supplementary Material). Additionally, the reviewer suggests to the authors that a criterion like the following: 'the orientation of Class1 provides better access to the AFM tip to the imaging of the appropriate zones of proteins for the analysis', or similar, should be used instead of that of 'the most abundant population'.

As suggested by the reviewer, we revised the justification of the selection criterium of the subgroup of Class1 orientations as follow.

"Since the orientation of Class1 provided better access to the HS-AFM tip to the imaging of the appropriate zones of accessory proteins for the analysis, we focused on Classs1 particles hereafter."

Minor Issues

1) The authors must detail how they generated the pseudo-AFM images from the alphafold results and the possible software packages they utilized

We described how to generate the pseudo-AFM images in the revised method section as follow.

"The PDB file of Alphafold2 predicted Slh1 structure (AF-P53327-F1-model_v4) was downloaded from the AlphaFold Protein Structure Database (https://alphafold.ebi.ac.uk). Based on the N-terminal (3-217 aa) deleted Slh1 structural model, the pseudo AFM image was constructed by a simple collision simulation between the probe and the center position of the protein atom, assuming the AFM probe to be a cone with a tip radius of 1.0 nm and an opening angle of 10 degrees, and ignoring the radius of the protein atom. The pseudo-AFM images were created using the laboratory-made analysis software FalconViewer based on Igor Pro-9 (WaveMetrics)."

2) Line 226. The authors write that Cue3 'engages the RQT complex at the right position', the term 'right position' is vague. The authors should clarify to the reader the meaning of 'right position'.

We have revised "at the right position" to "near the proximal ubiquitin site of uS10".

3) The authors show in their hs-afm movies that the Rqt4 domain explores the area surrounding the Slh1. This finding is key for the model the authors propose. For the analysis of the motion of the Rqt4 domain, the authors correctly measure the distance between the center of mass of the Slh1 and the end of the Rqt4. It is found that the Rqt4 explores a radial distance that reaches 20-40 nanometres. Nevertheless, the authors do not provide any information on the angular positions that the Rqt4 explores; it is not clarified whether the Rqt4 explores a certain range of angles with respect Slh1 in preference or not, such information would be of interest for the assessment of the probabilities of binding of Rqt4 to the K63-Ubi chain. This information could be shown in the form of radial plots that would substitute the ones of Fig.4b and Fig.4d, and/or in the form of an image of the zone of exploration that would be obtained using for instance the maximum filter for stacks available in the ImageJ; please find below an sample of a modified Fig.4 showing the maximum filter images of the movies 6 and 7 that are titled 'Maximum height from all frames'.

According to the reviewer's request, we added the 2D plot of the center position of Cue3 and the most distant position of Rqt4 in the revised Figure 4 f and g, respectively. These results provided information on the angular position of Cue3 and Rqt4 explorers. We have now described this as follow.

"To further analyze the 2D distribution of the accessory proteins of the RQT complex, we plotted the center position of Cue3 and the most distant position of Rqt4 in all frames on the HS-AFM images of Slh1/Cue3 and Slh1/Rqt4, respectively, indicating that the searchable range of Rqt4 was expanded by the IDRs, whereas the movable range of Cue3 was limited **(Fig. 4f-g)**."
